# MYCN and PRC1 cooperatively repress docosahexaenoic acid synthesis in neuroblastoma via ELOVL2

**DOI:** 10.1186/s13046-019-1492-5

**Published:** 2019-12-19

**Authors:** Yi Ding, Jie Yang, Yawen Ma, Tengteng Yao, Xingyu Chen, Shengfang Ge, Lihua Wang, Xianqun Fan

**Affiliations:** 1 Department of Ophthalmology, Ninth People’s Hospital, Shanghai JiaoTong University School of Medicine, Shanghai, 200011 China; 2 Shanghai Key Laboratory of Orbital Diseases and Ocular Oncology, Shanghai, 200011 China

**Keywords:** Neuroblastoma, Docosahexaenoic acid metabolism, MYCN, ELOVL2

## Abstract

**Background:**

The MYCN amplification is a defining hallmark of high-risk neuroblastoma. Due to irregular oncogenes orchestration, tumor cells exhibit distinct fatty acid metabolic features from non-tumor cells. However, the function of MYCN in neuroblastoma fatty acid metabolism reprogramming remains unknown.

**Methods:**

Gas Chromatography-Mass Spectrometer (GC-MS) was used to find the potential target fatty acid metabolites of MYCN. Real-time PCR (RT-PCR) and clinical bioinformatics analysis was used to find the related target genes. The function of the identified target gene ELOVL2 on cell growth was detected through CCK-8 assay, Soft agar colony formation assay, flow Cytometry assay and mouse xenograft. Chromatin immunoprecipitation (ChIP) and Immunoprecipitation-Mass Spectrometer (IP-MS) further identified the target gene and the co-repressor of MYCN.

**Results:**

The fatty acid profile of MYCN-depleted neuroblastoma cells identified docosahexaenoic acid (DHA), an omega-3 polyunsaturated fatty acid with anti-tumor activity, significantly increased after MYCN depletion. Compared with MYCN single-copy neuroblastoma cells, DHA level was significantly lower in MYCN-amplified neuroblastoma cells. RT-PCR and clinical bioinformatics analysis discovered that MYCN interfered DHA accumulation via ELOVL fatty acid elongase 2 (ELOVL2) which is a rate-limiting enzyme of cellular DHA synthesis. Enforced ELOVL2 expression in MYCN-amplified neuroblastoma cells led to decreased cell growth and counteracted the growth-promoting effect of MYCN overexpression both in vitro and vivo. ELOVL2 Knockdown showed the opposite effect in MYCN single-copy neuroblastoma cells. In primary neuroblastoma, high ELOVL2 transcription correlated with favorable clinical tumor biology and patient survival. The mechanism of MYCN-mediated ELOVL2 inhibition contributed to epigenetic regulation. MYCN recruited PRC1 (Polycomb repressive complex 1), catalysed H2AK119ub (histone 2A lysine 119 monoubiquitination) and inhibited subsequent ELOVL2 transcription.

**Conclusions:**

The tumor suppressive properties of DHA and ELOVL2 are repressed by the MYCN and PRC1 jointly, which suggests a new epigenetic mechanism of MYCN-mediated fatty acid regulation and indicates PRC1 inhibition as a potential novel strategy to activate ELOVL2 suppressive functions.

## Background

The MYC family of transcription factors, including MYC, MYCN, and MYCL, are the most commonly altered oncogenes in cancer [1, 2]. MYC mediates global metabolic reprogramming to match the enhanced demand for anabolic metabolites in tumor cells [3]. MYC enhances glucose intake and metabolism [4, 5], promotes the conversion of glutamine to glutamate and oxaloacetate/pyruvate to aspartate/alanine [6, 7], accelerates fatty acid biosynthesis [8, 9]. Similarly, recent studies have shown that tumors with an amplified MYCN gene, which is considered the most robust prognostic marker indicating poor outcome for neuroblastoma patients, enhanced glutamine transport and increased glutaminolysis for increased tumor cell growth [10–14]. However, the function of MYCN in global metabolic reprogramming is controversial. Some studies have shown that there is little or no correlation between MYCN amplification and glycolysis [15, 16], while no reports so far studied the relationship between MYCN and fatty acid metabolism. MYCN plays a critical role in neuroblastoma tumor initiation, aggressiveness and resistance to chemotherapy [17–19]. Collectively, the potential regulatory role of MYCN in metabolic reprogramming, especially fatty acid metabolism, should be comprehensively investigated to pave the way for novel therapies.

Alterations in fatty acid (FA) metabolism in cancer cells are increasingly being recognized. FAs are the primary source of energy storage, are the components of membrane phospholipids and generate signalling molecules [20]. Of particular interest within the FA family are polyunsaturated fatty acids (PUFAs), because PUFAs metabolism are highly activated in the neuron system [21]. As the main PUFA in neuron phosphatidylserine, DHA (docosahexaenoic acid, C22:6n-3), an omega-3 PUFAs, promotes neuronal survival and neurogenesis in the brain [22, 23]. DHA and its mediator synaptamide increases phosphatidylserine synthesis, and enhances neuronal differentiation, neurite growth, synaptogenesis and synaptic function [24–27]. Furthermore, numerous experimental evidences demonstrate that DHA exerts potent anti-tumor effects on colorectal, breast, bladder, prostate, oesophagus and glioblastoma cancers [28–30]. DHA level in most cancer tissues were found to be lower than those in normal tissues, and associated with a reduced risk of breast, prostate, colon and renal cancers [31, 32].

Interestingly, neuroblastoma initiates from proliferating early neural crest-derived progenitor cells unable to differentiate due to mistakes occurring during embryonal neuroendocrine or sympathoadrenal development [33–35]. Recent evidence also indicated that MYCN is involved in the acquisition and maintenance of pluripotency in neural stem cells [36]. Although there is no reported correlation between MYCN and DHA, the existing evidence suggests that both MYCN and DHA associate with neuroblastoma initiation and development [37, 38], and the relationship between these two factors should be comprehensively elucidated to develop novel treatments.

Here, we discovered that MYCN inhibition lead to significant DHA accumulation and upregulated its synthesis enzyme ELOVL2 in neuroblastoma cell lines. We assessed the expression of ELOVL2 in primary neuroblastomas and neuroblastoma cell lines and unravelled its tumor suppressor activity. Furthermore, we elucidated the upstream epigenetic repressive regulation of ELOVL2 through MYCN and PRC1 complex-mediated histone ubiquitination in MYCN-amplified neuroblastoma. The repression of ELOVL2 by the MYCN and PRC1 complex indicates PRC1 complex inhibition as a potential novel strategy to activate ELOVL2 tumor suppressive functions.

## Materials and methods

### Cell culture

The BE(2)-C, IMR-32 and SK-N-AS cell lines were obtained from the SIBS (Cell Resource Center, Shanghai Academy of Life Sciences, Chinese Academy of Sciences). High-throughput multiplex cell contamination testing was used to monitor cell line infections [39]. High-throughput SNP-based assays were used to validate cell line authenticity [40]. The BE(2)-C, IMR-32 and SK-N-AS cell lines were cultured in DMEM (Gibco) supplemented with 10% FBS (Thermo Fisher Scientific) and 1% non-essential amino acids (NEAAs; Sigma-Aldrich) at 37 °C and 5% CO2.

### siRNAs, plasmids and establishing stable cells

siRNA oligos targeting BMI1, RING1A, RING1B and the negative control siRNA were purchased from GenePharma and were transfected using Lipofectamine 2000 (Life Technologies) at a concentration of 50 nM. All siRNAs used are summarized in Table 1. The two shRNA sequences targeting MYCN and ELOVL2 were cloned into the pGIPZ lentivirus vector (System Biosciences). The CDS sequence of human MYCN, ELOVL2 and SREBP1 were generated by PCR and cloned into the CMV-MCS-EF1-ZsGreen1-PGK-Puro vector (Addgene). Transduction and viral infection were performed as previously described [41].

**Table 1:** Primer, siRNA and shRNA used in this study

Primer	Sequence of forward primer	Sequence of reverse primer
MYCN	ACTGTAGCCATCCGAGGACA	TCGGAAGCAGAAACAGTCCC
FADS1	GTTATCCAGCGAAAGAAGTGGG	CCAATAGTGGCACATAAGTGAGG
FADS2	GACCACGGCAAGAACTCAAAG	GAGGGTAGGAATCCAGCCATT
ELOVL1	TTATTCTCCGAAAGAAAGACGGG	ATGACATGCACGGAAGAGTTTAT
ELOVL2	GGAAGCTGACATCCGGGTAG	TCCAGTTCAAGACACACCACC
ELOVL5	CTATTGTCCCGCGATTGGCT	CAGCAGTGTGAGTCCAAGGT
ELOVL7	AGATGCTGATCCAAGAGTTGAAG	GCTGTGGGTGACCGTGAATA
SLC27A2	GTGCTGCACTACTGATTGGC	CCCAGTGCCAGTCTCACTTT
SLC27A3	ACCCTGTCTGACCCACTGTA	GGATCAGCTCCAGCCACATT
SREBF1	ACAGTGACTTCCCTGGCCTAT	GCATGGACGGGTACATCTTCAA
BMI1	AGTTTCCACTCTGCCTTCAGC	CATTGTCTCGCCCCGATCTC
RING1A	CATTGTCTCGCCCCGATCTC	GATTGCTGTTTCCCCTCGGT
RING1B	CGGGGAGAGGCGATGCTATT	TACCCAAAGCCTTCACACCAC
ELOVL2 Promoter	ATCAGTTCGGATAACGGCCC	TAGAAGCGCAGGCTCTAGGA
siRNA sequence		
BMI1-siRNA-1	CCUAAUACUUUCCAGAUUGAU tt	AUCAAUCUGGAAAGUAUUACGG tt
BMI1-siRNA-2	CGGAAAGUAAACAAAGACAAA tt	UUUGUCUUUGUUUACUUUCCG tt
RING1A-siRNA-1	GCCCUGAUCUCUAAGAUCUAU tt	AUAGAUCUUAGAGAUCAGGGC tt
RING1A-siRNA-2	AGACGAGGUAUGUGAAGACAA t t	UUGUCUUCACAUACCUCGUCU tt
RING1B-siRNA-1	GCCAGCAUCAACAAGCACAAU tt	AUUGUGCUUGUUGAUCCUGGC tt
RING1B-siRNA-2	GGAGUGUUUACAUCGUUUU tt	AAAACGAUGUAAACACUCC tt
shRNA sequence		
MYCN-shRNA-1	CCGGGCCAGTATTAGACTGGAAGTTCTCGAGAACTTCCAGTCTAATACTGGCTTTTTGAATTC	
MYCN-shRNA-2	CCGGCTGAGCGATTCAGATGATGAACTCGAGTTCATCATCTGAATCGCTCAGTTTTTGAATTC	
ELOVL2-shRNA-1	CCGGGGTGCTTTGGTGGTACTATTTCTCGAGAAATAGTACCACCAAAGCACCTTTTTGAATT	
ELOVL2-shRNA-2	CCGGTATGTTTGGACCGCGAGATTCCTCGAGGAATCTCGCGGTCCAAACATATTTTTGAATT	

### Lipid extraction and GC-MS

For lipid extraction, 1 × 10^6^ cells were collected and re-suspended in 250 μl PBS and 100 μl deuterated internal standards. Then, the suspended cell solution was mixed with 500 μl methanol and 25 μl 1 N HCl. A bi-phasic solution was formed by the addition of 1.5 ml of isooctane to the above solution. The bi-phasic solution was vigorously mixed for 30 s and separated into two phases by centrifugation at 3000 rpm for 5 min. The upper isooctane phase containing the free fatty acid (FFA) fraction was collected and evaporated to dryness. For GC-MS, an Agilent 6890 N gas chromatograph equipped with an Agilent 7683 autosampler (Santa Clara) was utilized and operated along with an Agilent 5975 mass selective detector in negative ion mode using NCI with methane as the reagent gas.

### Elisa

A DHA ELISA Kit (purchased from Cloud-Clone Corp) was used to measure the DHA concentration. Then, 50 μL of dilutions of the standard, blank and cell lysates were added into the appropriate wells, and 50 μL of Detection Reagent A was added to each well immediately. The plate was incubated for 1 h at 37 °C. The solution was aspirated and washed with 350 μL of 1X Wash Solution to each well 3 times. Then, 100 μL of Detection Reagent B working solution was added and incubated for 30 min at 37 °C. The solution was aspirated and washed with 350 μL of 1X Wash Solution to each well 3 times. Next, 90 μL of Substrate Solution was added to each well and incubated for 15 min at 37 °C, followed by the addition of 50 μL of Stop Solution to each well. The liquid turned yellow after the addition of Stop solution. The microplate reader was immediately used to conduct measurements at 450 nm.

### Cell growth assay

A CCK-8 colorimetric assay was used to measure the cell growth and viability, as described previously [42]. Briefly, 2 × 10^3^ cells per well were seeded into a flat-bottomed 96-well microtiter plate and incubated with either solvent control (0.1% DMSO) or various concentrations of DHA. At 0, 24, 48, and 72 h, spent medium was carefully removed and 100 μL DMEM containing 10% CCK-8 solution (TaKaRa) was added to each well and incubated for 3 h at 37 °C with 5% CO2, the relative cell number was recorded by a Benchmark Microplate Reader at 450 nm (Bio-Rad Laboratories).

### Cell cycle analysis

The cell cycle profile of the treated BE(2)-C, IMR-32 and SK-N-AS cells was analysed by flow cytometry. A total of 1 × 10^5^ treated cells per well were seeded into a 6-well plate dish and incubated overnight. On the day of analysis, the cells were permeabilized with 70% ethanol at 4 °C for 30 min. Subsequently, the cells were treated with propidium iodide (PI) and analysed by flow cytometry (FACSCanto flow cytometer; BD Biosciences) for cell cycle distribution using the ModFit LT V3.0 software (Verity Software House, Topsham).

### Western blot analysis

Cells were lysed for Western blotting in RIPA buffer (Cell Signaling Technology) with complete protease inhibitor cocktail (Roche). For the detection of histone H3 acetylation levels, cells were lysed in SDS lysis buffer (62.5 mM Tris-HCl, 2% SDS, 10% (v/v) glycerol, 1 mM DTT and complete protease inhibitor cocktail). The following antibodies were used: anti-MYCN (B8.4.B, sc-53,993, Santa Cruz), anti-ELOVL2 (EPR11880, ab176327, Abcam), anti-monoubiquitin H2A (Lys119) (ABE569, Millipore), anti-BMI1 (6964, Cell Signaling Technology), anti-RING1A (2820, Cell Signaling Technology), anti-RING1B (5694, Cell Signaling Technology), anti-SREBP1 (sc-13,551, Santa Cruz Biotechnology), and anti-ß-actin (clone AC-15, Sigma-Aldrich).

### RNA extraction and qRT-PCR

The RNeasy Mini Kit (Qiagen) was used to extract total RNA from cell lines or snap-frozen xenograft tissue. The TaKaRa PrimeScript RT reagent kit was used to transcribe cDNA for qRT-PCR analysis. The relative gene expression was measured using SYBR Green qPCR Master Mix (Perkin-Elmer Applied Biosystems) on an ABI Flex 6 thermal cycler (Perkin-Elmer Applied Biosystems) and normalized to the averaged expression of 18S mRNA. The primers used in qRT-PCR are listed in Table 1. Data were analysed using Applied Biosystems QuantStudio™ Real-Time PCR Software, and changes in expression were calculated using the ΔΔCT method for cell lines.

### ChIP

A total of 1 × 10^6^ cells were collected and lysed for ChIP in buffer containing 50 mM Tris-HCl, pH 8.1, 1% SDS, 10 mM EDTA and complete protease inhibitor cocktail (Roche), then sonicated to obtain 200–1000 bp DNA fragments. ChIP was performed according to the ChIP Assay Kit (Millipore) protocol. The following antibodies were used: anti-MYCN (B8.4.B, sc-53,993, Santa Cruz), anti-acetyl-histone H3 (Lys9) (7–352, Millipore), anti-trimethyl-histone H3 (Lys4) (07–473, Millipore), anti-monoubiquitin H2A (Lys119) (ABE569, Millipore), anti-BMI1 (6964, Cell Signaling Technology), anti-RING1A (2820, Cell Signaling Technology), anti-RING1B (5694, Cell Signaling Technology), anti-SREBP1 (sc-13,551, Santa Cruz Biotechnology), normal mouse IgG (sc-2025, Santa Cruz) and normal rabbit IgG (sc-2027, Santa Cruz). Primers for specific qRT-PCR amplification of the ELOVL2 promoter region were: forward: 5′-ATCAGTTCGGATAACGGCCC-3′, reverse: 5′- TAGAAGCGCAGGCTCTAGGA-3′.

ChIP sequencing data of ELOVL2 promoter interactions with MYCN and H3K4me3 in NGP, SK-N-BE and SK-N-SH NB cell lines were achieved from Gene Expression Omnibus (GSM2113529, GSM2113519 and GSM2308437), and the analysis was performed online in the Cistrome platform (http://cistrome.org).

### Co-immunoprecipitation (co-IP) assays

Co-IP assays were performed to identify the proteins. Briefly, 1 × 10^6^ cells were collected and lysed for IP-MS in 300 μL buffer containing 1% NP40, 150 nM NaCl, 50 mM Tris-Cl (pH 7.2), 1 mM EDTA nuclear extracts (NE) and complete protease inhibitor cocktail (Roche). For Co-IP using antibodies, before being added to the cell lysates, the antibodies were incubated with Protein A/G Magnetic Beads (Millipore) for 3 h at 4 °C. Then, the crosslinked Protein A/G Magnetic Beads (Millipore) were added to the cell lysates directly and incubated overnight at 4 °C. The magnetic beads were washed with IP wash buffer and collected. The protein complexes were eluted from the beads by 50 mM glycine (pH 2.8) and analysed by MS.

### Animal experiments

BE(2)-C cells were transfected for 96 h with empty, MYCN and/or ELOVL2 vectors in culture, and SK-N-AS cells were transfected for 72 h with the negative control or shRNA targeting MYCN and/or ELOVL2 vectors in culture; then, 2 × 10^6^ viable cells suspended in 200 μl Basement Matrigel (BD Biosciences) were subcutaneously implanted in the flanks of 4-week-old female nude mice (*n* = 5 per study group). Tumor size was measured with a calliper every 3 or 4 days. After 24 days, the animals were sacrificed, and the tumor volume was calculated by π/6(s1 × s2 × s2), where s1 was the largest tumor diameter, and s2 was the smallest tumor diameter. Experiments conformed to the regulatory standards and were approved by the local ethics committee.

### Statistical analysis

Kaplan-Meier analyses and comparison of ELOVL2 expression between different patient subgroups for the 496 patients neuroblastoma cohort were performed online in the R2 platform (http://r2.amc.nl), and the resulting survival curves, box plots and *P* values (log-rank test) were downloaded. The results of the cell culture experiments were compared using the one-sample t-test in GraphPad Prism version 5.0 (GraphPad Software Inc., La Jolla, CA) unless otherwise indicated. P values below 0.05 were considered significant.

## Results

### MYCN negatively regulates DHA synthesis via ELOVL2

**Fig. 1:**
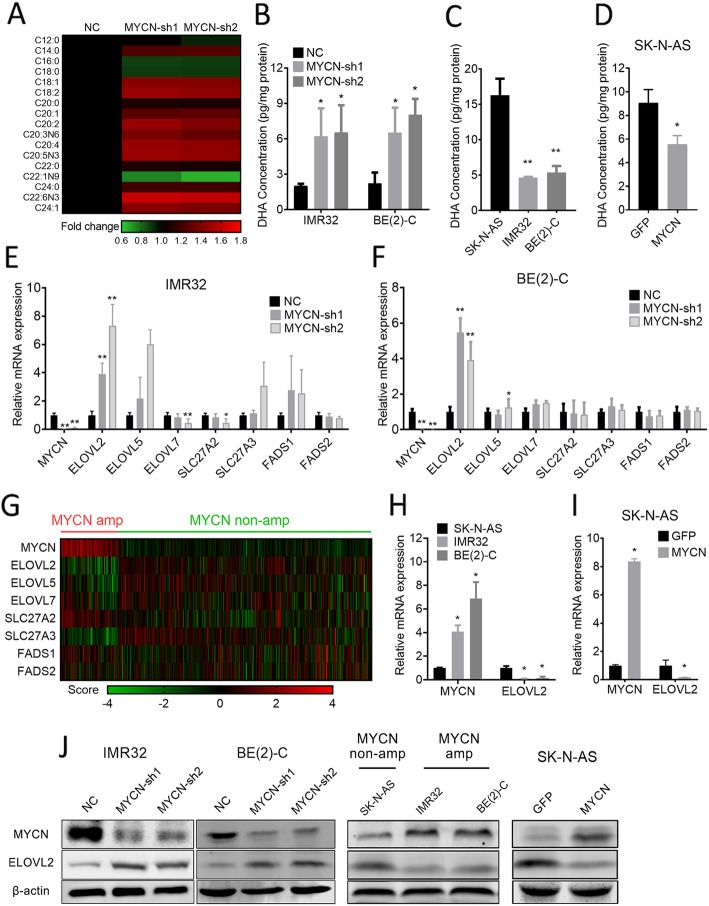
MYCN RNAi strongly upregulated DHA and ELOVL2 in neuroblastoma cell lines. **a** Heatmap representation of FA profiling indicates significantly (*P* < 0.05) up- (red) or downregulated (green) FAs in BE(2)-C cells treated for 72 h with the lentivirus expressing 2 MYCN shRNA or the negative control. **b** IMR32 and BE(2)-C cells were treated as described in (**a**). The change in DHA concentration was validated using ELISA. **c** DHA concentration of MYCN-amplified cell lines (IMR32 and BE(2)-C) and MYCN single-copy cell lines SK-N-AS were measured by ELISA. **d** The DHA concentration of SK-N-AS cells stably expressing the GFP control or MYCN was measured by ELISA. **e** and **f** IMR32 and BE(2)-C stably expressing negative control or MYCN shRNA. The change in DHA transport and synesis-related mRNA expression was measured by qRT-PCR (fold-change over negative control ± SD). **g** RNA expression pattern of ELOVL2 with MYCN amplified verse MYCN nonamplified in a cohort of 476 neuroblastoma. **h** MYCN and ELOVL2 mRNA expression of MYCN-amplified cell lines (IMR32 and BE(2)-C) and MYCN single-copy cell lines (SK-N-AS) were measured by qRT-PCR (fold-change over SK-N-AS ± SD). **i** MYCN and ELOVL2 mRNA expression of SK-N-AS cells stably expressing GFP control or MYCN were measured by qRT-PCR (fold-change over GFP control ± SD). **j** Using an MYCN and ELOVL2 antibody with the ß-actin loading control. MYCN RNAi efficiency and the change in ELOVL2 expression was validated by Western blots (left). The difference of MYCN and ELOVL2 between MYCN-amplified cell lines (IMR32 and BE(2)-C) and MYCN single-copy cell lines (SK-N-AS) was validated by Western blots (middle). MYCN overexpression efficiency and the change in ELOVL2 expression were validated by Western blots (right). *P < 0.05; **P < 0.01

To identify the potential role of MYCN in FA metabolism regulation, we first used GC-MS to profile the medium- and long-chain FA landscape after MYCN depletion in the MYCN-amplified neuroblastoma cells IMR32. IMR32 cells were infected with the lentivirus expressing 2 shRNAs targeting MYCN or the negative control for 72 h before GC-MS profiling. MYCN depletion resulted in significant upregulation of various types of FAs (Fig. 1a), of which DHA was the most strongly upregulated with a 1.6- to 1.61-fold induction. ELISA analysis validated that DHA was dramatically upregulated (3.1- to 3.2- fold in IMR32 and 2.9- to 3.6- fold in another MYCN-amplified neuroblastoma cell line, BE(2)-C cells (Fig. 1b). Considering that the strongest DHA induction by MYCN depletion occurred in MYCN-amplified cells, we speculated that the endogenous DHA content are different in neuroblastoma cell lines with different MYCN genomic statuses. As shown in Fig. 1c, the MYCN-amplified cell lines BE(2)-C and IMR32 expressed distinctly lower DHA levels than SK-N-AS cells, which maintained a single MYCN copy. Furthermore, enforced MYCN expression reduced endogenous DHA levels in MYCN-nonamplified SK-N-AS cells (Fig. 1d). We next tested the direct influence of DHA on cell growth by a CCK-8 assay. After DHA treatment, IMR32 and BE(2)-C cells exhibited lower proliferation rates in a DHA concentration-dependent manner (Additional file 1: Figure S1A). Meanwhile, the cell cycle profiles of IMR32 and BE(2)-C cells were examined by flow cytometry. DHA treatment caused cell cycle arrest at the G0/G1 phase (Additional file 1: Figure S1B). Moreover, DHA treatment reduced the colony formation of two cells in soft agar (Additional file 1: Figure S1C). Given that, MYCN suppresses DHA transporter or synthesis to favor tumor cell growth.

To identify the metabolic activity causally involved, eight known DHA synthesis and transporter genes were detected by qPCR in BE(2)-C and IMR32 cells, which expressed low levels of MYCN after shRNA-mediated knockdown using two different shRNAs. MYCN depletion most strongly induced ELOVL2 expression in both cells, resulting in a 3.9- to 7.3-fold induction in IMR32 cells and a 3.9- to 5.5-fold induction in BE(2)-C cells (Fig. 1e and f). ELOVL7 increased 2.2 (ns)- to 6.0-fold in IMR32 cells and 1.4- to 1.5-fold in BE(2)-C cells (Fig. 1 **e** and **f**). Meanwhile, depletion of MYCN slightly downregulated SLC27A2 and FADS2 in IMR32 cells and did not affect the expression of ELOVL4, ELOVL5, SLC27A3 or FADS1 (Fig. 1 **e** and **f**). In line with the mRNA expression level after MYCN depletion, the protein level of ELOVL2 was also upregulated in both neuroblastoma cell lines (Fig. 1j). Importantly, by using the SEQC-498 cohort, we performed a differential RNA expression analysis of the MYCN-amplification and nonamplification neuroblastoma tumors. Of all seven known DHA synthesis and transporter genes, the decrease of ELOVL2 expression was mostly significant in MYCN amplified neuroblastoma (Fig. 1g). We further reanalysed RNA-sequencing data from a series of neuroblastomas cohort and draw the same conclusion that ELOVL2 expression was significantly correlated with MYCN status (Additional file 2: Figure S2). In line with clinical tumor samples, the MYCN-amplified cell lines BE(2)-C and IMR32 expressed lower ELOVL2 expression than MYCN-nonamplified cell lines SK-N-AS cells (Fig. 1h), and the protein level of ELOVL2 was vilified in Fig. 1j. Enforced MYCN expression reduced ELOVL2 expression in MYCN-nonamplified SK-N-AS cells (Fig. 1i), and the protein level of ELOVL2 was vilified in Fig. 1j. In conclusion, our data showed that MYCN represses DHA synthesis via ELOVL2 inhibition.

### ELVOL2 is critical for DHA accumulation and proliferation inhibition in neuroblastoma cells

**Fig. 2:**
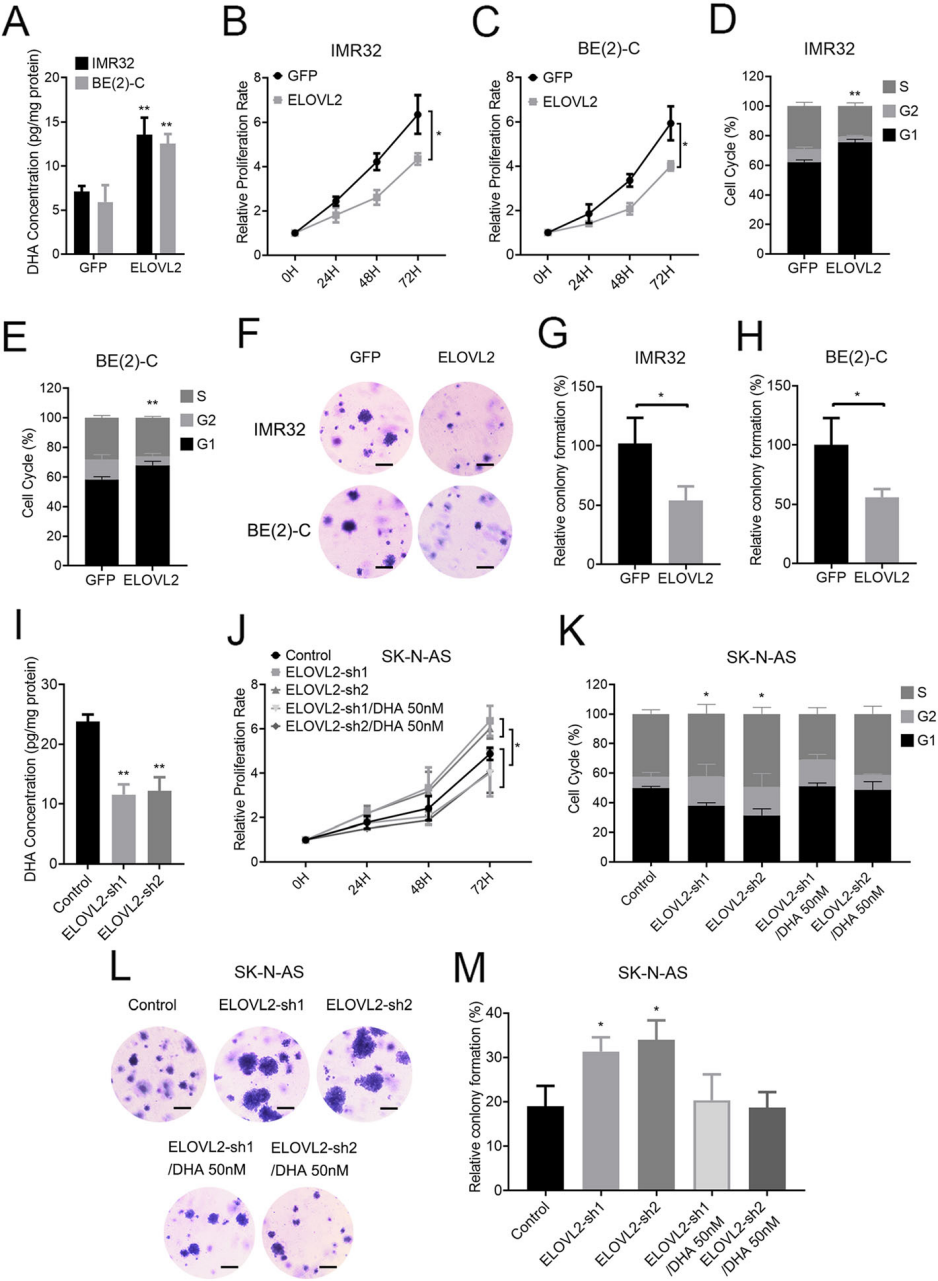
ELOVL2 inhibits cell proliferation in vitro. **a** Relative DHA concentrations of IMR32 and BE(2)-C cells stably expressing GFP or ELOVL2 were measured by ELISA (fold-change over GFP control ± SD). **b** and **c** The proliferation rate of IMR32 and BE(2)-C cells stably expressing GFP or ELOVL2 was assayed using CCK-8 and a spectrophotometer at OD450. **d** and **e** IMR32 and BE(2)-C cells stably expressing GFP or ELOVL2 were stained with PI for 72 h, and the change in the cell cycle was measured by flow cytometry. **f** IMR32 and BE(2)-C stably expressed GFP or ELOVL2. Colony growth in soft agar is shown for representative cultures after staining with crystal violet. Scale bar, 25 μm. **g** and **h** The results of the soft agar assays of (**f**) are presented as bar graphs of the mean number of colonies (±SD) forming. **i** Relative DHA concentration of SK-N-AS cells stably expressing the negative control or ELOVL2 shRNA was measured by ELISA (fold-change over negative control ± SD). **j** SK-N-AS cells stably expressing the negative control or ELOVL2 shRNA were treated with DHA 50 nM. The proliferation rate was assayed using CCK-8 and a spectrophotometer at OD450. **k** SK-N-AS cells stably expressing the negative control or ELOVL2 shRNA were stained with PI for 72 h, and the change in the cell cycle was measured by flow cytometry. **l** SK-N-AS cells stably expressed the negative control or ELOVL2 shRNA. Colony growth in soft agar is shown for representative cultures after staining with crystal violet. Scale bar, 25 μm. **m** The results of the soft agar assays of (L) are presented as bar graphs of the mean number of colonies (±SD) forming. *P < 0.05; **P < 0.01

To test the biological effects of ELOVL2 in MYCN-amplified neuroblastoma cells, ELOVL2 was sustainably overexpressed in MYCN-amplified BE(2)-C and IMR32 cells by a lentivirus expressing ELOVL2. Transfection efficacy was validated by assessing the expression of ELOVL2 by qPCR and western blot (Additional file 3: Figure S3). Enforced ELOVL2 expression increased the DHA content up to 1.4- and 1.5-fold in IMR32 and BE(2)-C cells, respectively (Fig. 2a), and reduced the proliferation rate of both cells (Fig. 2b and c). The cell cycle profiles of IMR32 and BE(2)-C were examined by flow cytometry. Enforced ELOVL2 expression caused cell cycle arrest at the G0/G1 phase, accompanied by a decrease in the percentage of cells in the S phase, especially in IMR32 cells (Fig. 2d and e). Moreover, enforced ELOVL2 expression reduced the colony formation of these two cell lines in soft agar (Fig. 2f, g and h).

SK-N-AS Cells with a single copy of MYCN endogenously express higher ELOVL2 levels. Hence, we knocked down ELOVL2 in SK-N-AS cells with two different shRNAs. Transfection efficacy was validated by assessing the expression of ELOVL2 by qPCR (Additional file 3: Figure S3). ELOVL2 knockdown decreased the DHA content (Fig. 2i), increased the proliferation rate (Fig. 2j), decreased cell cycle arrest at the G0/G1 phase (Fig. 2k), and increased colony formation in soft agar (Fig. 2l and m); re-supplementation with 50 nM DHA partly counteracted the pro-proliferation effect induced by ELOVL2 depletion. Moreover, re-supplementation with other kinds of PUFA, including 50 nM LA (linoleic acid), ALA (α-Linolenic acid), AA (Arachidonic acid) or DPA(n-6) (Docosapentaenoic Acid n-6) did not counteracted the pro-proliferation effect induced by ELOVL2 depletion, and re-supplementation with 50 nM EPA (Eicosapentaenoic Acid) partly counteracted the pro-proliferation effect induced by ELOVL2 depletion (Additional file 4: Figure S4). Therefore, ELOVL2 possesses anti-tumor properties in neuroblastoma cells via DHA synthesis.

### MYCN transcriptionally regulates ELOVL2 expression via PRC1-mediated H2AK119ub in neuroblastoma cells

**Fig. 3:**
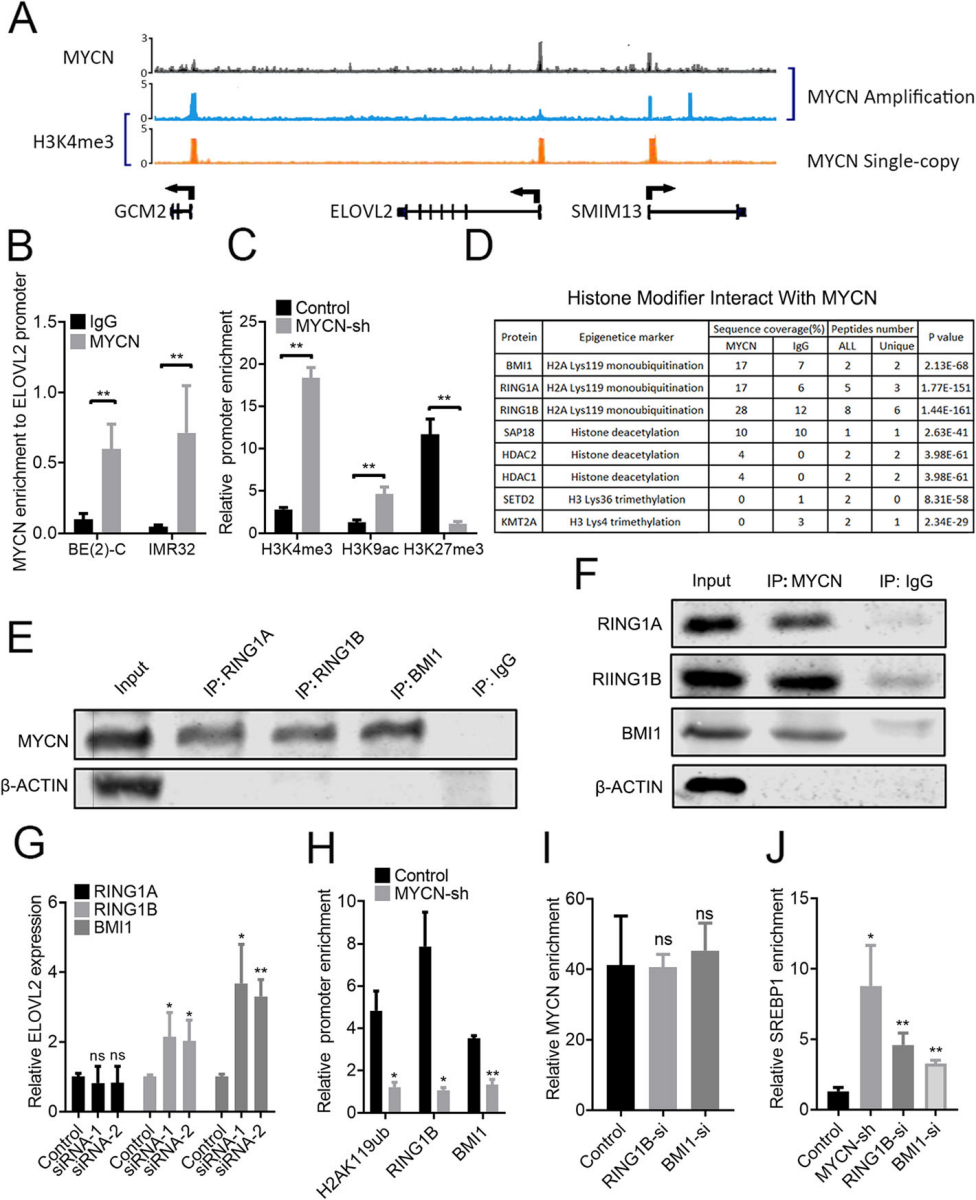
MYCN recruits the PRC1 complex to the ELOVL2 promoter region and mediates ELOVL2 inhibition by H2AK119ub. **a** ChIP-Seq analysis from MYCN-amplified cell lines (NGP and BE(2)-C) and MYCN single-copy cell lines (SK-N-SH) using antibodies detecting MYCN (black bar) and H3K4me3 (blue and orange bar) superimposed on the ELOVL2 cluster genomic organization. **b** ChIP analysis in the IMR32 and BE(2)-C cell lysates showing MYCN recruitment to the ELOVL2 promoter region. **c** ChIP analysis in BE(2)-C cells stably expressing the negative control or ELOVL2 shRNA showing reduced H3K4me3 and H3K9ac enrichment, and increased H3K27me3 enrichment to the ELOVL2 promoter region. **d** BE(2)-C cell lysates immunoprecipitated with antibodies against MYCN were analysed using mass spectrometry to detect potential binding partner proteins of MYCN. The PRC1 complex consisting of BMI1, RING1A and RING1B was detected. **e** and **f** Western blots of BE(2)-C cell lysates coimmunoprecipitated with antibodies against MYCN, BMI1, RING1A and RING1B shown with the IgG control antibody and 1% of the lysate input to assess the PRC1-MYCN protein interaction. **g** qRT-PCR showing relative ELOVL2 mRNA expression (fold-change over negative control ± SD) in BE(2)-C cells 48 h after BMI1, RING1A or RING1B siRNA treatment. **h** ChIP analysis showing relative ELOVL2 promoter enrichment of H2AK119ub, RING1B and BMI1 in BE(2)-C cells treated as in (B). **i** ChIP analysis showing relative ELOVL2 promoter enrichment of MYCN in BE(2)-C cells 48 h after BMI1 or RING1B siRNA treatment. **j** ChIP analysis showing relative ELOVL2 promoter enrichment of SREBP1 in BE(2)-C cells treated as in (**b**) and (**i**). *P < 0.05; **P < 0.01

Protein binding profiles from the Gene Expression Omnibus ChIP-seq database revealed that MYCN could bind to the ELOVL2 promoter in the MYCN-amplified cell line NGP, and histone 3 lysine 4 tri-methylation (H3K4me3) was enriched at the ELOVL2 promoter region in the MYCN single-copy cell line SK-N-SH but not in the MYCN-amplified cell line BE(2)-C (Fig. 3a). Further ChIP-qPCR analysis confirmed the Cistrome database results, which showed abundant enrichment of MYCN at the ELOVL2 promoter in BE(2)-C and IMR32 cells (Fig. 3b), increased H3K4me3 and H3K9ac, and reduced H3K27me3 upon MYCN knockdown in BE(2)-C cells (Fig. 3c). Next, we aimed to decipher events occurring at the ELOVL2 promoter region. An immunoprecipitation-mass spectrometry (IP-MS) approach was used to identify interactive proteins with MYCN, which identified a small set of transcription factors, including RING1A, RING1B and BMI1, three major components of polycomb repressive complex 1 (PRC1) (Fig. 3d). To verify the results of IP-MS, we tested whether the MYCN, RING1A, RING1B and BMI1 proteins physically cooperate by Western blots in BE(2)-C cells. IP with an antibody against MYCN showed that the enrichment of RING1A, RING1B and BMI1 were significantly higher in the anti-MYCN group than in the IgG control group (Fig. 3e). IP with an antibody against RING1A, RING1B and BMI1 showed an increased enrichment of MYCN above that in the IgG control (Fig. 3f). To verify PRC1 components involved in the transcriptional repression of ELOVL2, we transiently knocked down RING1A, RING1B and BMI1 using two different siRNAs to eliminate nonspecific and off target effects in BE(2)-C cells, and we then assessed ELOVL2 expression. Transfection efficacy was validated by qPCR (Additional file 4: Figure S4C). Depletion of RING1A did not affect ELOVL2 expression. RING1B and BMI1 depletion induced ELOVL2 expression 2.0- to 2.1-fold and 3.3- to 3.7-fold 72 h after knockdown, respectively (Fig. 3g). To test whether the PRC1 complex was recruited to this ELOVL2 promoter region and led to H2AK119ub (histone 2A lysine 119 monoubiquitination), we performed ChIP-qPCR. RING1B and BMI1 were recruited to this region, and H2AK119ub enrichment was elevated (Fig. 3h). To analyse whether MYCN is required for PRC1 complex-mediated H2AK119ub at the ELOVL2 promoter, we performed ChIP-qPCR. Stable MYCN knockdown in BE(2)-C cells decreased the recruitment of RING1B and BMI1 to the ELOVL2 promoter by ~ 90% and ~ 60%, respectively, reducing H2AK119ub enrichment by ~ 80% (Fig. 3h). RING1B or BMI1 knockdown did not decrease the enrichment of MYCN to the ELOVL2 promoter (Fig. 3i). SREBP1 was a known transcription factor that activated ELOVL2 expression [43], which was further verified by ChIP-qPCR, a luciferase assay and a Western blot in the neuroblastoma cells SK-N-AS (Additional file 5: Figure S5). MYCN, RING1B and BMI1 knockdown increased the recruitment of SREBP1 to the ELOVL2 promoter by 7-, 3.5- and 2.5-fold, respectively (Fig. 3j). In conclusion, MYCN recruits the PRC1 complex, co-occupies the ELOVL2 promoter, mediates H2AK119ub and suppresses its transcription in neuroblastoma cells.

### ELVOL2 inhibits tumor progression in vivo and predicts favorable survival

**Fig. 4:**
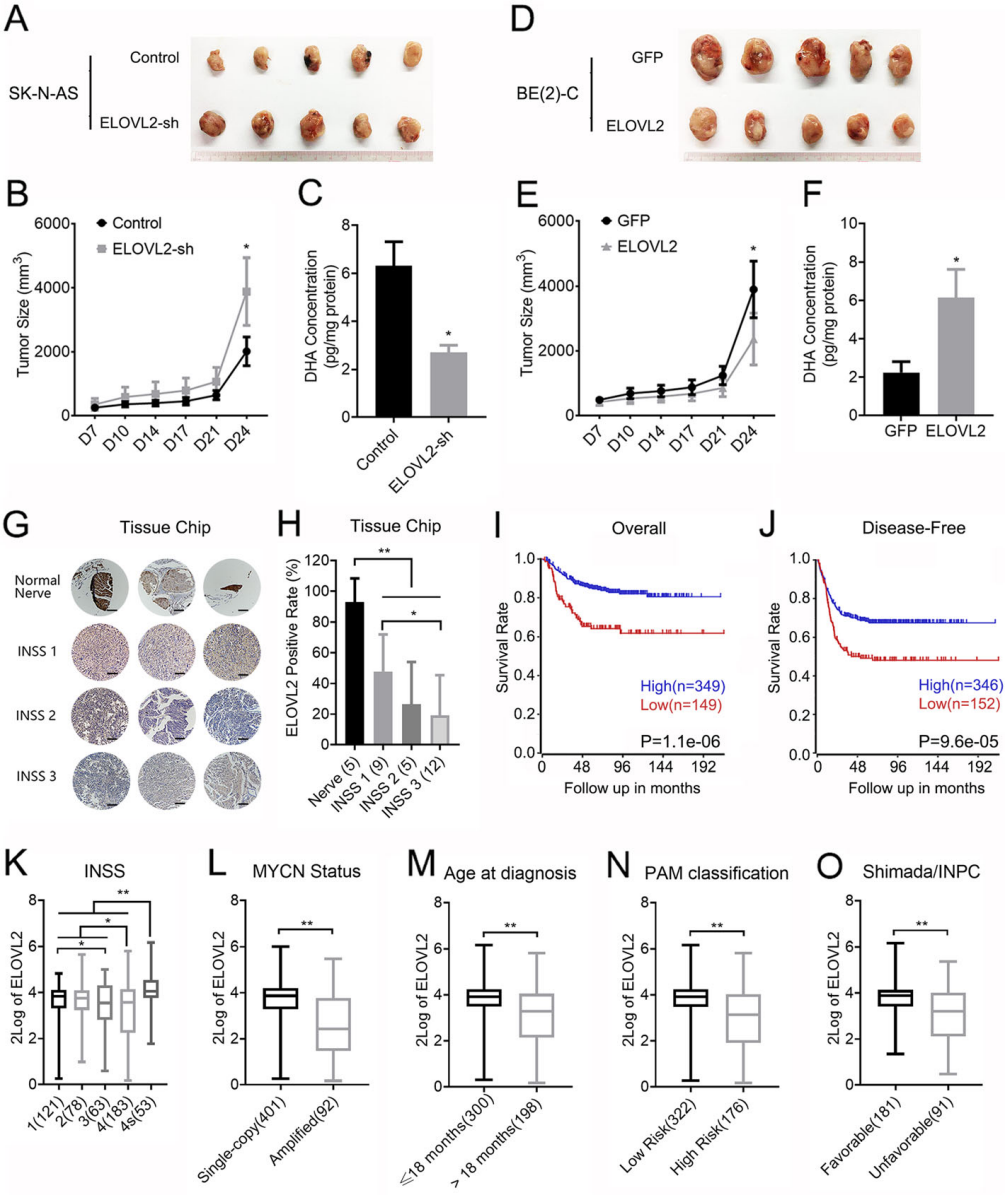
ELOVL2 suppresses tumor growth in vivo and correlates with favorable neuroblastoma patient survival. **a**, **b** and **c** SK-N-AS cells stably expressing negative control or ELOVL2 shRNA were injected subcutaneously into nude mice (*n* = 5 for each group). Tumors were compared (**a**) at the end of the experiment. Tumor growth curves (**b**) were measured starting at 7 days after inoculation. The DHA concentration of tumors (**c**) was measured by ELISA. **d**, **e** and **f** BE(2)-C cells stably expressing ELOVL2 or the GFP control were injected subcutaneously into nude mice (n = 5 for each group). Tumors were compared (**d**) at the end of the experiment. Tumor growth curves (**e**) were measured starting at 7 days after inoculation. The DHA concentration of tumors (**f**) was measured by ELISA. **g** Representative IHC analysis of ELOVL2 protein expression in normal nerve tissues and neuroblastoma specimens of different INSS stages (1–3) is shown. Scale bar, 100 μm. **h** The results of IHC analysis of ELOVL2 expression are presented as bar graphs of the mean number of the ELOVL2-positive rate. **i** and **j** Overall (**i**) and disease-free (**j**) Kaplan-Meier curves with univariate analyses for patients with low versus high ELOVL2 mRNA expression in a cohort of 476 tumors. **k**, **l**, **m**, **n** and **o** High-level ELOVL2 mRNA expression in neuroblastomas correlates with favorable clinical features, including low INSS stage (**k**), single-copy MYCN status (**l**), ≤18 months age at diagnosis (**m**), low risk of PAM classification (**n**) and favorable Shimada/INPC (**o**) in a cohort of 476 tumors. *P < 0.05; **P < 0.01

Analysis of mouse xenografts using SK-N-AS cells that stably expressed shRNA targeting ELOVL2 showed that knockdown of ELOVL2 significantly enhanced tumor volume (Fig. 4a and b), mass (Additional file 6: Figure S6A) and decreased the DHA content (Fig. 4c) compared to those in the nontargeting control group. On the other hand, analysis of mouse xenografts using BE(2)-C cells that stably expressed ELOVL2 showed that compared to the GFP control group, the enforced ELOVL2 expression group had a significantly reduced tumor volume (Fig. 4d and e), mass (Additional file 6: Figure S6B) and an increased DHA content (Fig. 4f).

To further explore the clinical feature of ELOVL2 in neuroblastoma, we assessed whether ELOVL2 is differentially expressed in primary tumors. By IHC analysis of a tissue chip, we compared the difference in ELOVL2 protein expression in normal nerve tissues and neuroblastoma specimens with different international neuroblastoma staging system (INSS) stages. ELOVL2 expression is relatively lower in neuroblastoma than in normal nerve tissues and negatively correlated with INSS stage (Fig. 4g and h). The detailed patient information was provided in Additional file 7: Table S1. We reanalysed RNA-seq data from a cohort of 496 neuroblastomas [44]. Kaplan-Meier analysis showed that high-level ELOVL2 expression in tumors correlated both with favorable overall and event-free patient survival (Fig. 4i and j). Furthermore, at the transcriptional level, high-level ELOVL2 expression also significantly correlated with established clinical and molecular markers for favorable tumor biology, including INSS 1, 2 or 4S stage disease (Fig. 4k), Single-copy MYCN status (Fig. 4l), age at diagnosis ≤18 months (Fig. 4m), a low-risk tumor transcriptional profile defined by principal access method (PAM) analysis (Fig. 4n) and favorable Shimada/INPC tumor histology (Fig. 4o). Taken together, ELOVL2 inhibits neuroblastoma growth in vivo and could be regarded as a favorable marker.

### MYCN regulates tumor progression via ELOVL2 repression in vitro and in vivo

**Fig. 5:**
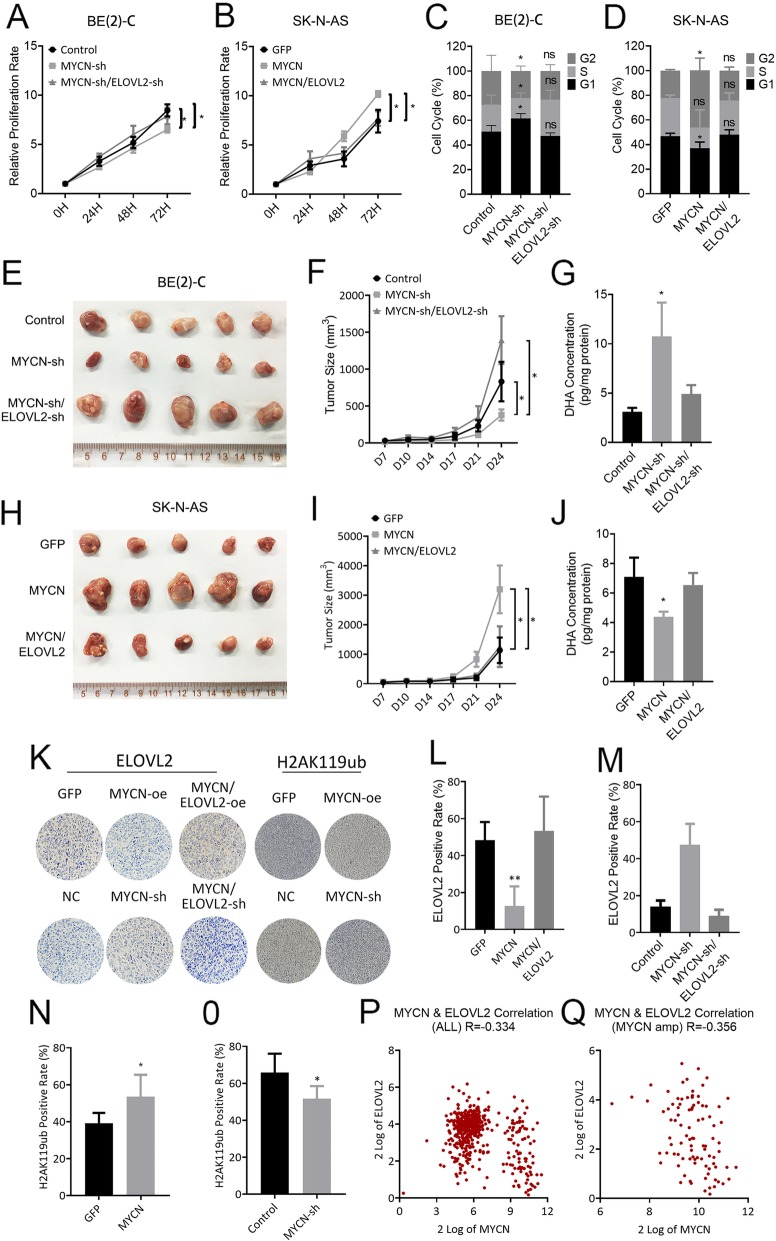
MYCN promotes tumor growth by repressing ELOVL2 in vitro and vivo. **a** and **b** BE(2)-C cells stably expressing the negative control or MYCN shRNA were further infected with a lentivirus expressing ELOVL2 shRNA. SK-N-AS cells stably expressing GFP or MYCN were further infected with a lentivirus expressing ELOVL2. The BE(2)-C (**a**) and SK-N-AS (**b**) cell proliferation rate was determined using CCK-8 and a spectrophotometer at OD450. **c** and **d** BE(2)-C (**c**) and SK-N-AS (**d**) cells were treated as described in (**a**). Cells were stained with PI for 72 h, and the change in the cell cycle was measured by flow cytometry. **e**, **f** and **g** BE(2)-C were treated as described in (**a**) and injected subcutaneously into nude mice (n = 5 for each group). Tumors were compared (**e**) at the end of the experiment. Tumor growth curves (**f**) were measured starting at 7 days after inoculation. The DHA concentration of tumors (**g**) was measured by ELISA. **h**, **i** and **j** SK-N-AS were treated as described in (**a**) and injected subcutaneously into nude mice (n = 5 for each group). Tumors were compared (**h**) at the end of the experiment. Tumor growth curves (**i**) were measured starting at 7 days after inoculation. The DHA concentration of tumors (**j**) was measured by ELISA. **k** Representative IHC analysis of ELOVL2 and H2AK119ub protein expression in SK-N-AS cells stably expressing GFP or MYCN (up) and BE(2)-C cells stably expressing the negative control or MYCN shRNA (low). **l**, **m**, **n** and **o** The results of IHC analysis of ELOVL2 and H2AK119ub protein expression are presented as bar graphs of the mean number of the ELOVL2 and H2AK119ub positive rate. **p** and **q** ELOVL2 expression significantly correlated with MYCN expression in ALL (P) and MYCN (Q) amplification clinical tumor samples from the SEQC database. *P < 0.05; **P < 0.01; ***P < 0.001

To further confirm the repression of ELOVL2 in MYCN-induced cell proliferation, we knocked down ELOVL2 in MYCN-depleted BE(2)-C cells, which resulted in re-increased cell proliferation (Fig. 5a) and cell cycle acceleration (Fig. 5c). Mouse xenograft results further showed that ELOVL2 knockdown again re-enhanced tumor growth (Fig. 5e and f), mass (Additional file 6: Figure S6C) and re-decreased the DHA content in tumors (Fig. 5g). In contrast, enforced ELOVL2 expression in MYCN-overexpressing SK-N-AS cells showed that enforced ELOVL2 expression diminished the induction effect of MYCN on cell proliferation (Fig. 5b) and cell cycle acceleration (Fig. 5d). Mouse xenograft results showed that enforced ELOVL2 expression diminished the promotion effect of MYCN on tumor growth (Fig. 5h and i), mass (Additional file 6: Figure S6D) and re-raised the DHA content in tumors (Fig. 5j). These results demonstrated that ELOVL2 is repressed, at least partly, for the regulation of cell proliferation by MYCN. By IHC analysis of mouse xenografts, we found that enforced MYCN expression downregulated the expression of ELOVL2 and upregulated the H2AK119ub level. In contrast, MYCN depletion upregulated the expression of ELOVL2 and downregulated the H2AK119ub level (Figs. 4k-o).

The expression level of ELOVL2 in neuroblastomas from the 496-tumor cohort was significantly correlated with MYCN status. In overall and MYCN-amplified neuroblastoma, ELOVL2 expression is significantly correlated with MYCN expression with a − 0.334 and − 0.356 R value, respectively (Fig. 5p and q).

## Discussion

Unlike the vague role of MYCN in FA metabolism, MYC was thought to play a key role in accelerating FA and cholesterol metabolism for sustainable tumor cell growth. MYC activates the expression of the enzymes ATP citrate lyase (ACLY), acetyl-CoA carboxylase alpha (ACACA), fatty acid synthase (FASN), and stearoyl-CoA desaturase (SCD), which are all involved in fatty acid synthesis from citrate [45]. In human tumor samples, a high level of MYC in prostate cancer specifically leads to enhanced lipid metabolism [46]. In triple-negative breast cancer, MYC overexpression correlates with an increased dependency on mitochondrial FA oxidation [3]. Currently, no researchers have reported the relationship between MYCN and FA metabolism. Hence, it remains to be elucidated whether the potential regulation of FA metabolism by MYCN in neuroblastoma is different from the MYC regulation in other tumor types.

Here, we identified DHA as the strongest upregulated FA after MYCN depletion and validated the tumor suppressive properties of DHA in highly malignant MYCN-amplified neuroblastoma cells. Increasing evidence has indicated the direct anti-tumor prosperity of DHA on various types of cancer cells, including neuroblastoma cells, in vitro. The mechanisms involved in the anticancer property of DHA are thought to proceed via their anti-inflammatory effects [47], their effects on the COX and LOX enzymes [48, 49], their effects on many transcription factors [50, 51], and their participation in various pathways, including activation of the Hippo pathway [52], apoptosis pathway [53] and autophagy pathway [54]. However, the regulation of DHA metabolism in tumor cells has rarely been discussed. Interestingly, mice fed a polyunsaturated fat diet enriched in DHA develop more preneoplastic foci in liver, indicating a tumor-specific DHA metabolism pattern in distinct tumor types [55]. Hence, further research on DHA metabolism is a prerequisite to fully exploiting the therapeutic application of DHA. We further unravelled that the DHA synesis enzyme ELOVL2 was remarkably upregulated after MYCN depletion and its tumor suppressive properties in this childhood malignancy both in vitro and in vivo. ELOVL2 encodes a transmembrane protein that controls the elongation of PUFA [56]. Studies have shown that ELOVL2 primarily controls the elongation process of PUFAs with 22 carbons to produce 24-carbon precursors for DHA (C22:6n-3) and DPA (C22:5n-6) formation [57, 58]. Further research in mice indicated that Elovl2 ablation led to more impairment of systemic DHA than DPA [57]. In the literature, the function of ELOVL2 in tumor cells remains unclear and controversial. For example, in hepatocellular carcinoma, a high level of ELOVL2 was significantly associated with poor hepatocellular carcinoma (HCC) prognosis [59]. In prostate cancer, ELOVL2 showed a notable upregulation in SPOP mutations that mediate drug resistance [60]. For patients with breast cancer, ELOVL2 can be hormonally upregulated by the estrogen receptor alpha (ERα), and this upregulation contributes to tumor development [61]. However, a single nucleotide polymorphism (SNP) at ELOVL2 rs3734398 was significantly associated with good prognosis in cutaneous melanoma [62]. Moreover, DNA methylation at ELOVL2 is statistically significantly associated with high-risk future breast and male colorectal cancer development [63]. ELOVL2 also participates in innate and adaptive immune regulation, which might weaken the effects of immunotherapies. For example, a low level of ELOVL2 is important for maintaining β-cell function [64]. Deletion of ELOVL2 in a mouse model leads to an increase in Th1 and Th17 T cells and a decrease in Foxp3+ regulatory T cells [65]. In our experiments, the data on ELOVL2 presented here identify that declined DHA synesis caused by MYCN-mediated ELOVL2 inhibition in neuroblastoma cells contributes to tumor aggressiveness.

The tumor suppressive function of ELOVL2 in preclinical neuroblastoma models prompted us to decipher the transcriptional regulation of ELOVL2. We conducted IP-MS using an MYCN antibody and found that polycomb repressive complex 1 (PRC1) physically cooperated with MYCN. PRC1 depletion caused an increase in ELOVL2 expression. PRC1 acts through histone H2A monoubiquitination at lysine 119, which is comprised of three main dominant RING finger E3 ligases: BMI1, RING1B and RING1A. BMI1 was originally identified as an oncogenic cooperation factor of MYC in murine lymphomagenesis [66]. Subsequent studies have further supported an oncogenic role for BMI1 in diverse human malignancies and associated BMI1 with tumor initiation and propagation, disease progression and poor prognosis [67, 68]. BMI1 is highly expressed in human neuroblastomas and neuroblastoma cell lines [69, 70]. Moreover, BMI1 was shown to cooperate with MYCN in the transformation of benign S-type neuroblastoma cells [71]. RING1B is highly expressed in a number of human malignancies, including prostate cancer, pancreatic ductal adenocarcinoma, ovarian cancer and urothelial bladder carcinoma [72–75]. Overexpression of RING1B has been associated with a poor prognosis for women with ovarian cancer and decreased survival times for patients with urothelial bladder carcinoma [74, 76]. Although there was no direct evidence showing that RING1B physically cooperates with MYCN, one study indicated that RING1B associates with other repressive complexes, including E2F-6.com-1, which is involved in the silencing of E2F and MYC-responsive genes in quiescent cells [77]. From our data, this new regulatory mechanism is promising because its upstream regulatory control involving MYCN and PRC1 is able to be disrupted with drugs reducing MYCN protein expression, possibly in combination with small molecular PRC1 inhibitors, such as PRT4165 [78] and PTC-209 [79]. Our studies also verified that SREBP1 is a transcriptional activator of ELOVL2 in neuroblastoma cells [43]. Interestingly, SREBP1 is an important regulator of lipogenesis and is thought to support tumor growth, such as breast tumors, renal cell carcinoma and glioblastoma [80–82]. However, clinical RNA sequencing data showed that high levels of SREBP1 expression in primary neuroblastomas correlate with clinical and molecular characteristics of a favorable tumor biology and indicates a favorable patient prognosis (data not shown). These findings suggest that SREBP1 can act as an oncogene or a tumor suppressor, depending on the tumor type.

## Conclusions

**Fig. 6:**
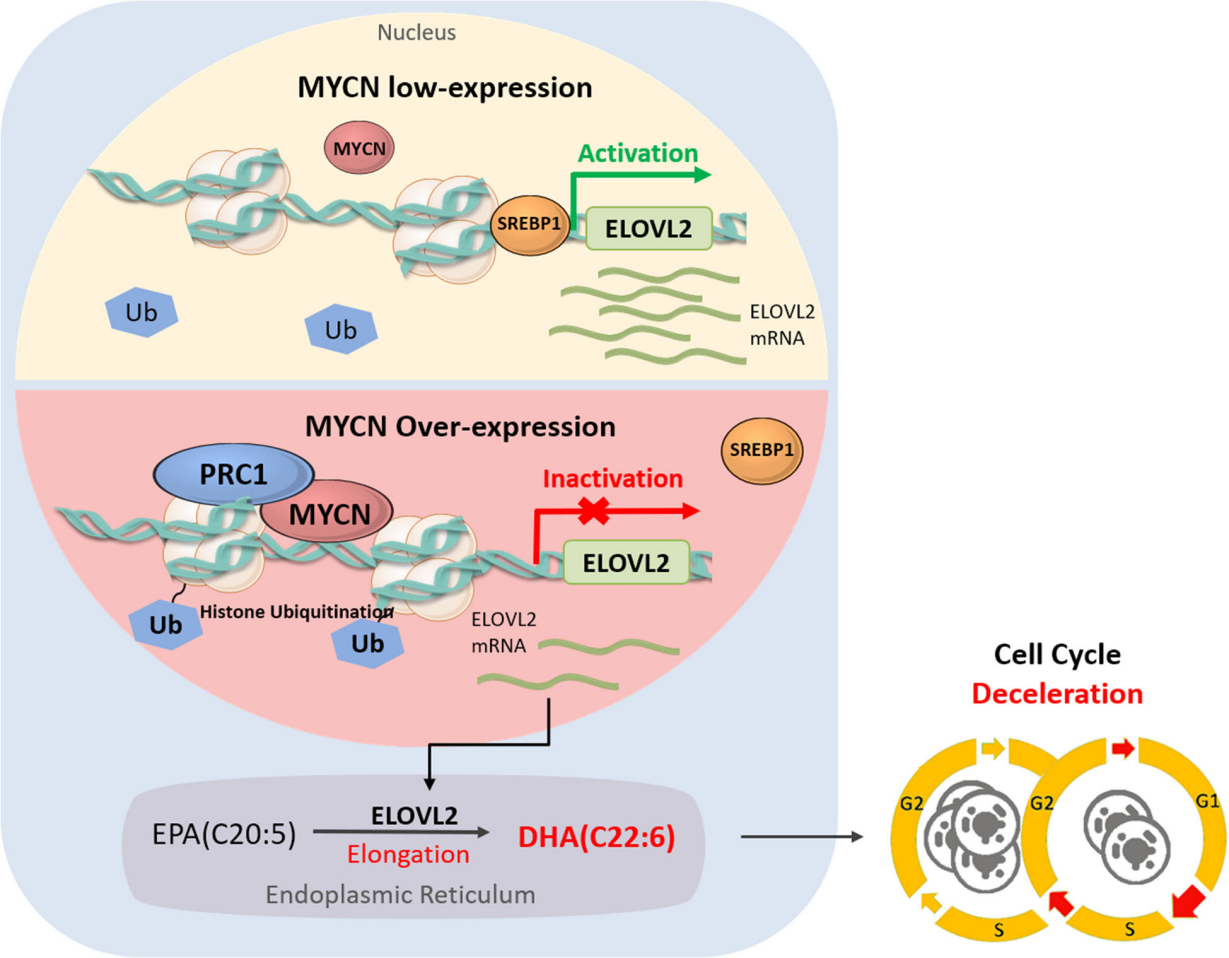
Schematic model of MYCN/PRC1-mediated ELOVL2 repression in neuroblastoma. MYCN and PRC1 are recruited in the same complexes to the ELOVL2 promoter region and repress ELOVL2 expression. The repressive epigenetic marker H2AK119ub is more enriched in MYCN-amplified neuroblastoma cells, and the ELOVL2 expression is decreased. The DHA concentration is correspondingly decreased, which finally lead to cell cycle acceleration

In conclusion, here, we report that DHA was the strongest FA responding to treatment with MYCN depletion. In primary neuroblastomas, high expression levels of ELOVL2, which encodes DHA synesis enzyme, correlated with a established favorable clinical and molecular characteristics of tumor biology and indicates a favorable patient survival. ELOVL2 inhibited cell proliferation and the colony formation of MYCN-amplified neuroblastoma cell lines and mice xenografts, demonstrating that ELOVL2 suppresses critical malignancy processes. ELOVL2 also responded to treatment with PRC1 siRNA, indicating the reversion of MYCN/PRC1-mediated transcriptional inhibition of ELOVL2 (modelled in Fig. 6). These data further our understanding of MYCN and PRC1 function in fatty acid metabolism, which also provides a new strategy for therapeutic intervention of high-risk MYCN-amplified neuroblastoma by jointly blocking MYCN and PRC1 activity.

## Supplementary information

**Additional file 1: Figure S1.** DHA inhibited neuroblastoma cell growth, cell cycle, and tumor growth in soft agar.

**Additional file 2: Figure S2.** RNA-seq results from one cohort of normal brain and four cohort of neuroblastoma.

**Additional file 3: Figure S3.** The ELOVL2 overexpression and RNAi efficiency.

**Additional file 4: Figure S4.** The influence of re-supplementation with different kinds of PUFA on cell proliferation after ELOVL2 depletion.

**Additional file 5: Figure S5.** SREBP1 up-regulated DHA content via ELOVL2.

**Additional file 6: Figure S6.** Mouse xenograft tumor weight.

**Additional file 7: Table S1.** Patient information of tissue chip.

## Data Availability

The datasets used and analyzed during the current study are available from the corresponding authors on reasonable request.

## Abbreviations

DHA: Docosahexaenoic acid
ELOVL2: ELOVL fatty acid elongase 2
H2AK119ub: Histone 2A lysine 119 monoubiquitination
MYCN: MYCN Proto-Oncogene, BHLH Transcription Factor
PRC1: Polycomb repressive complex 1
