# Supplementary figures and images for: MYCN and PRC1 cooperatively repress docosahexaenoic acid synthesis in neuroblastoma via ELOVL2
Source: J Exp Clin Cancer Res. 2019 Dec 19;38:498. doi: 10.1186/s13046-019-1492-5 (PMC6923955; doi:10.1186/s13046-019-1492-5)

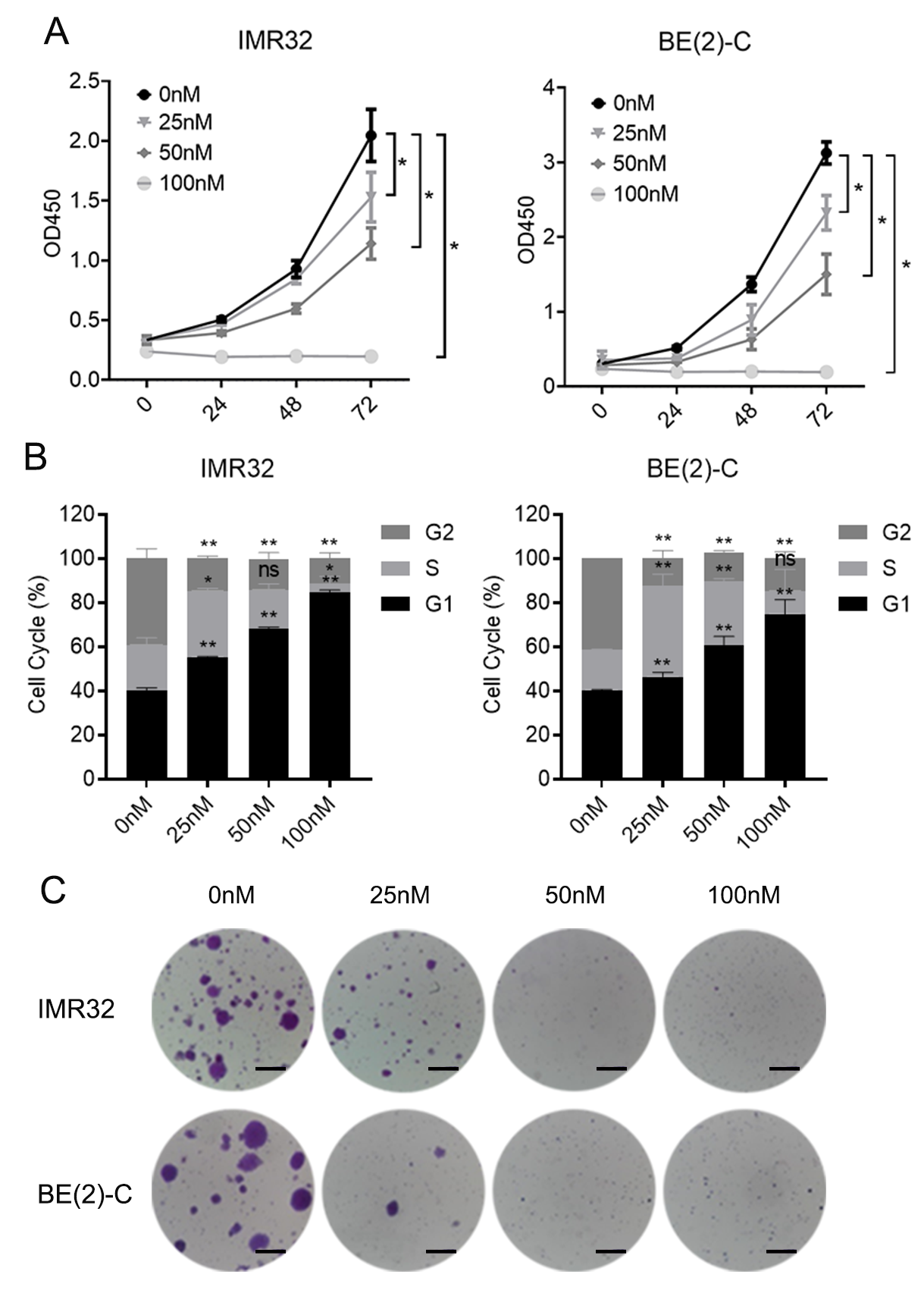

Supplement: Supplementary file 1 — Additional file 1: Figure S1. DHA inhibited neuroblastoma cell growth, cell cycle, and tumor growth in soft agar. [file 13046_2019_1492_MOESM1_ESM.tif]

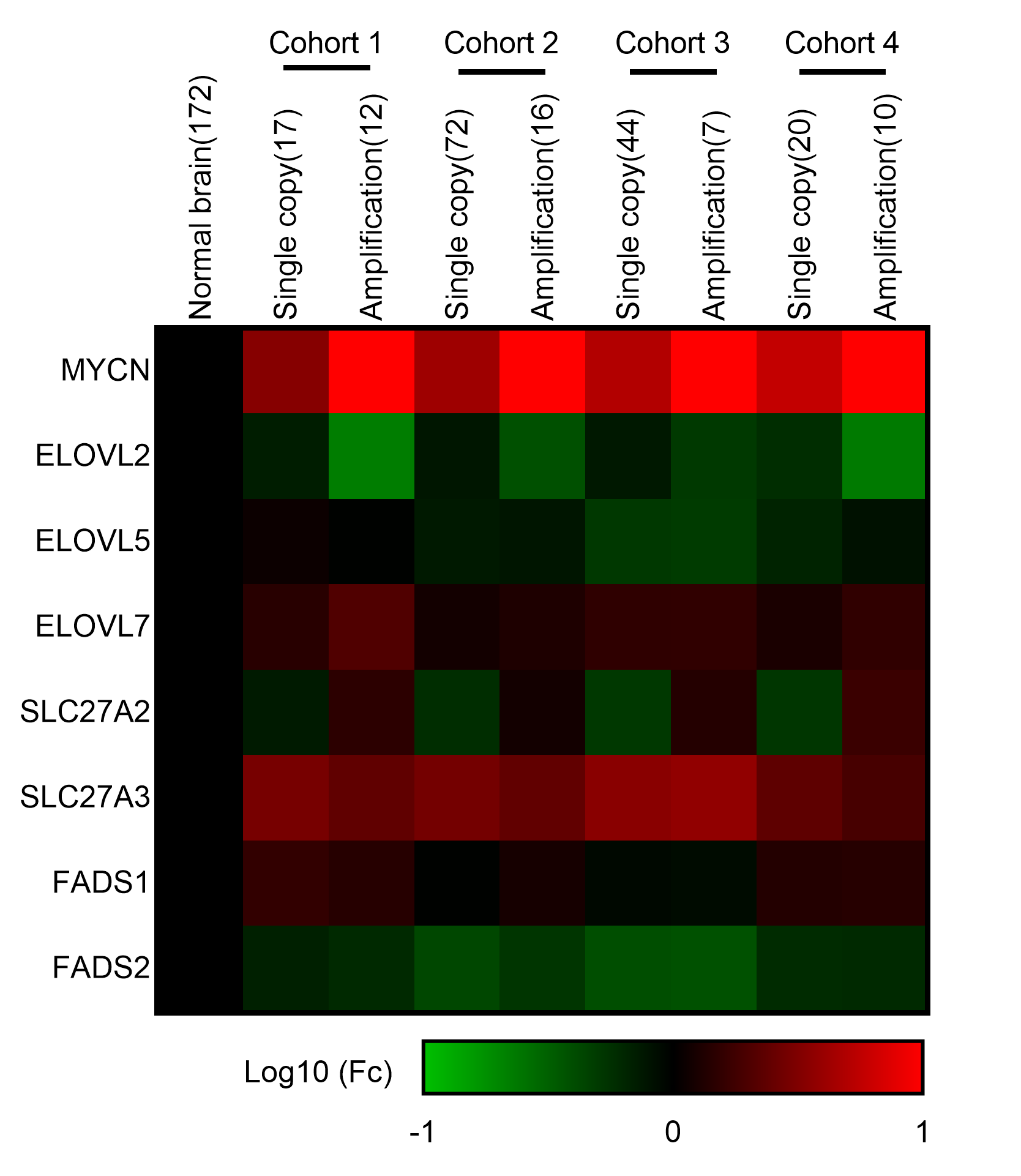

Supplement: Supplementary file 2 — Additional file 2: Figure S2. RNA-seq results from one cohort of normal brain and four cohort of neuroblastoma. [file 13046_2019_1492_MOESM2_ESM.tif]

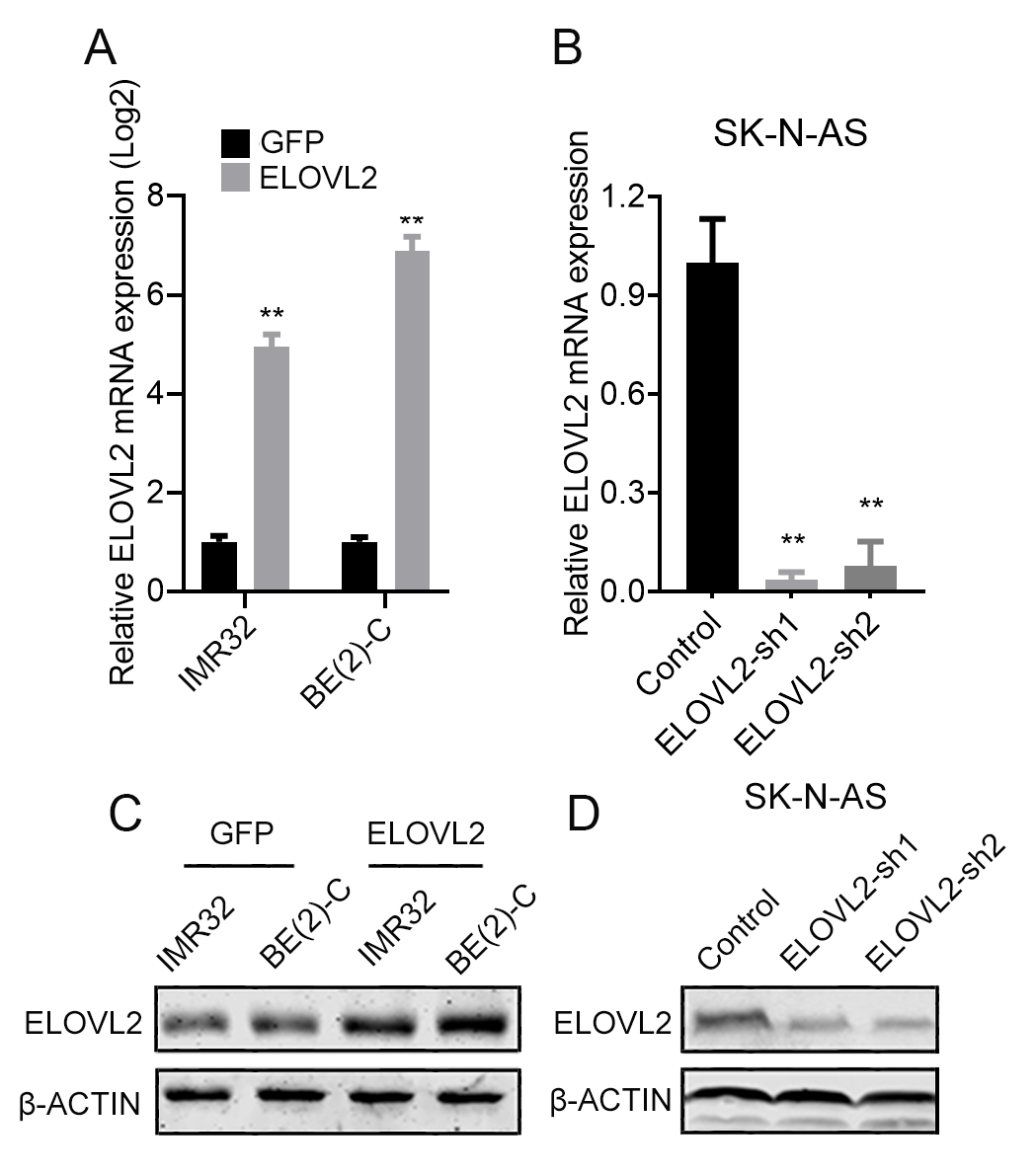

Supplement: Supplementary file 3 — Additional file 3: Figure S3. The ELOVL2 overexpression and RNAi efficiency. [file 13046_2019_1492_MOESM3_ESM.tif]

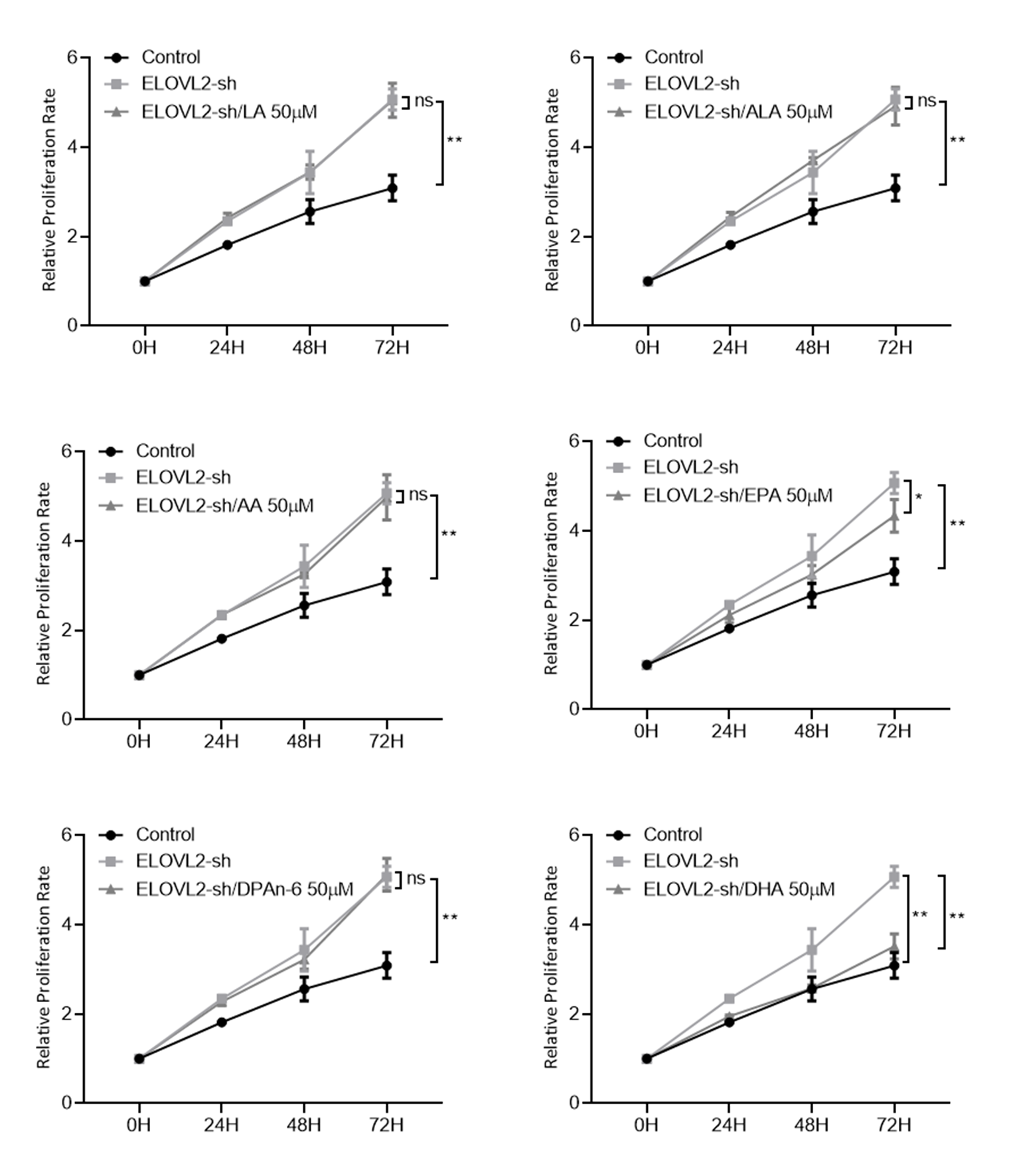

Supplement: Supplementary file 4 — Additional file 4: Figure S4. The influence of re-supplementation with different kinds of PUFA on cell proliferation after ELOVL2 depletion. [file 13046_2019_1492_MOESM4_ESM.tif]

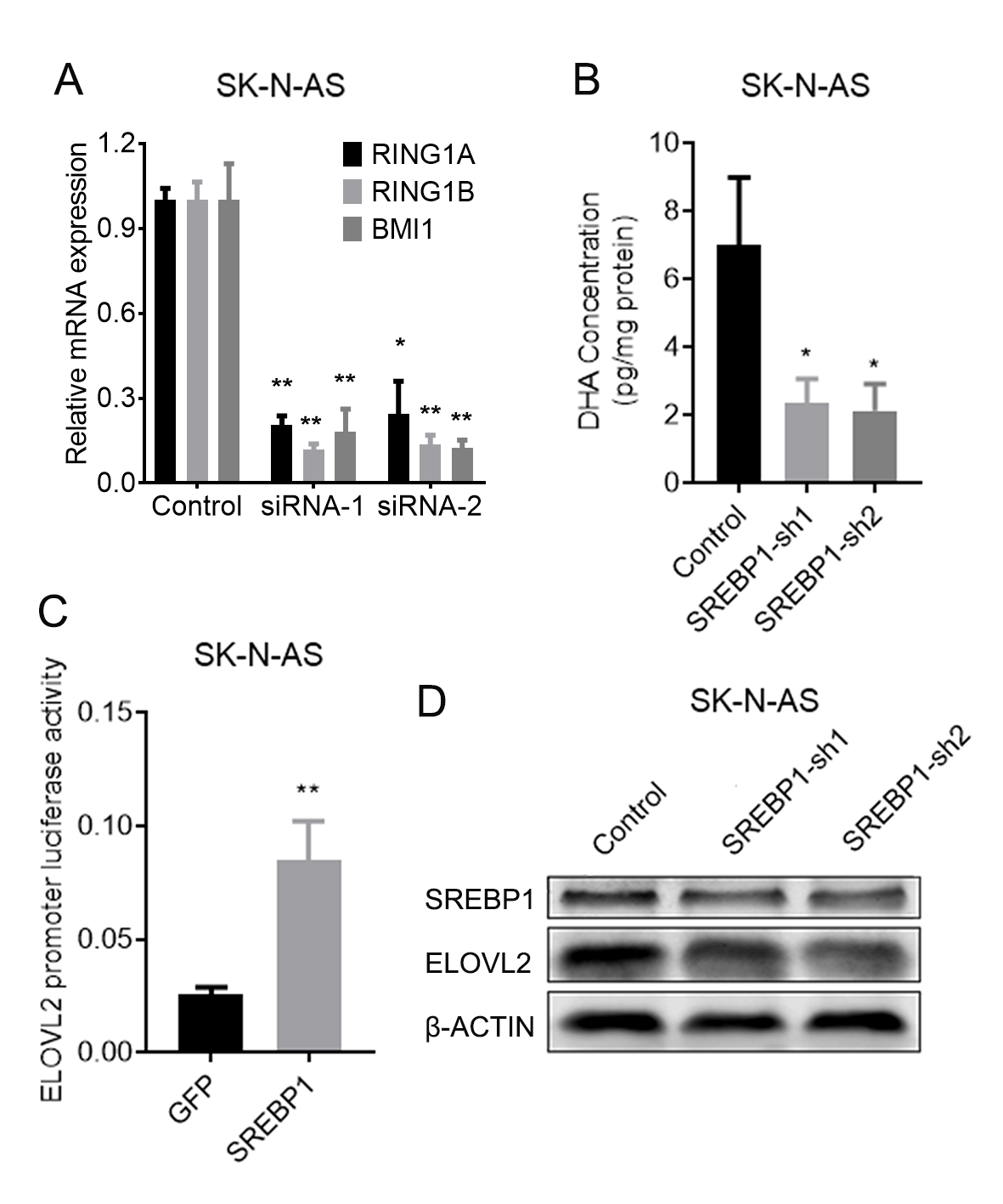

Supplement: Supplementary file 5 — Additional file 5: Figure S5. SREBP1 up-regulated DHA content via ELOVL2. [file 13046_2019_1492_MOESM5_ESM.tif]

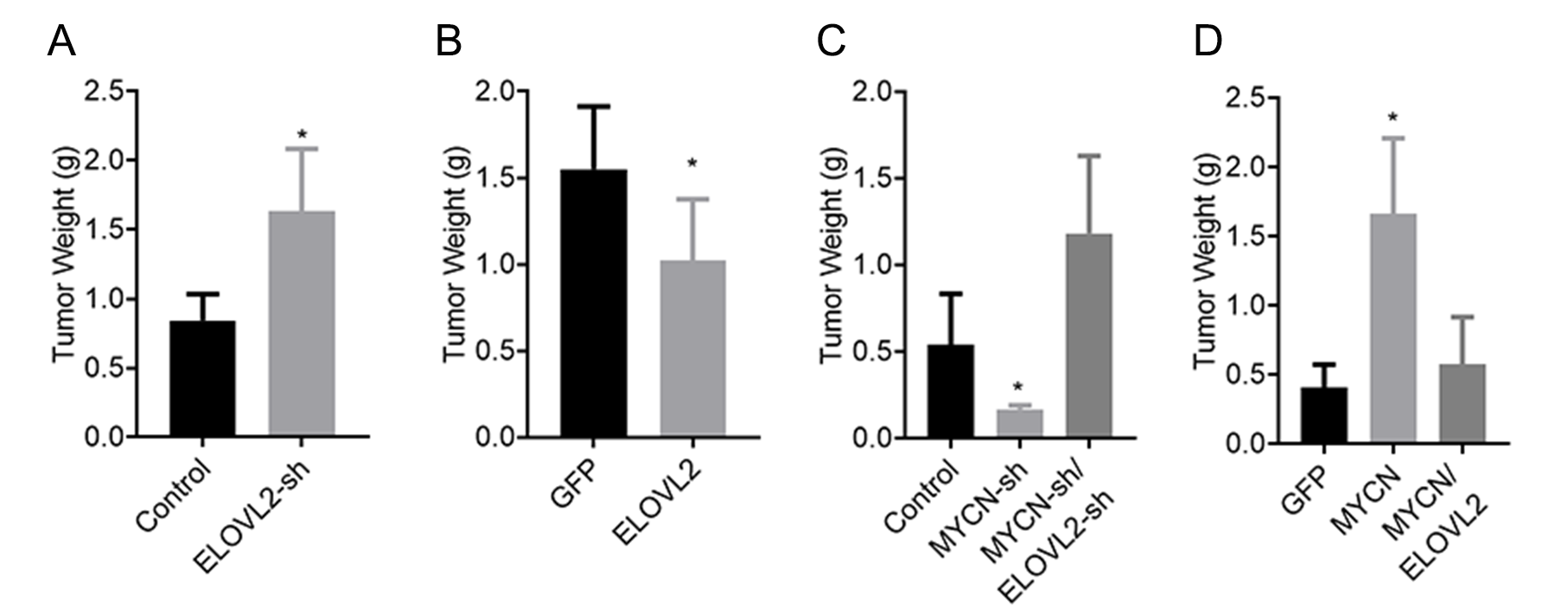

Supplement: Supplementary file 6 — Additional file 6: Figure S6. Mouse xenograft tumor weight. [file 13046_2019_1492_MOESM6_ESM.tif]
